# Miniaturization of Implantable Micro-Robot Propulsion Using a Wireless Power Transfer System

**DOI:** 10.3390/mi8090269

**Published:** 2017-09-01

**Authors:** Dongwook Kim, Karam Hwang, Jaehyoung Park, Hyun Ho Park, Seungyoung Ahn

**Affiliations:** 1 The Cho Chun Shik Graduate School for Green Transportation, Korea Advanced Institute of Science and Technology (KAIST), Daejeon 34141, Korea; dwkim88@kaist.ac.kr (D.K.); hwang8@kaist.ac.kr (K.H.); jaehyoung.park@kaist.ac.kr (J.P.); 2 Department of Electronic Engineering, the University of Suwon, Hwaseong 18123, Korea; hhpark@suwon.ac.kr

**Keywords:** micro-robot, biomedical devices, wireless power transfer, miniaturization, propulsion

## Abstract

This paper presents an efficient coil design for a mm-sized micro-robot which generates a propulsion force and torque and receives electrical energy using a wireless power transfer system. To determine the most efficient coil structures and produce propulsion and torque on the micro-robot, both helical and spiral coil modeling was conducted, and analytical formulations of the propulsion force and torque were derived for helical and spiral coil structures. Additionally, the dominant dimensional factors for determining propulsion and coil torque were analyzed in detail. Based on the results, an optimum coil structure for generating maximum force on the micro-robot was developed and is herein presented with dimensional analysis. Simulations and experiments were also conducted to verify the design, and good agreement was achieved. A 3-mm micro-robot that simultaneously generated a propulsion force and torque and received electrical energy via wireless power transfer was successfully fabricated using the proposed method and verified.

## 1. Introduction 

Recently, implantable micro-robots have been actively studied for medical applications. Micro-robots are particularly interesting for medical applications because of their tiny size, which allows them to potentially operate inside blood vessels [1,2,3]. Micro-robots have been proposed that could be used for the localized delivery of chemical and biological substances, and to operate using various forms of energy [2]. This provides a number of advantages over convention methods. For instance, by targeting drug delivery, it becomes possible to increase the concentration of a drug only in the region of interest, which reduces the risk of side effects in the rest of the body [2,3]. As interest in bio-medical implantable micro-robots increases, research on ways to control the movement of micro-robots is being pursued [4,5,6,7,8,9]. To control and propel micro-robots, most studies have adopted the approach of using a permanent magnet inside the micro-robot. This allows the motion of the micro-robot and its propulsion to be controlled by applying an external static magnetic field. However, this kind of micro-robot can perform only limited missions, such as drilling or moving [10,11,12,13,14,15,16,17]. 

To perform more complex and various missions with micro-robots, active circuits or elements that operate with electrical energy are required. To address these energy requirements, some researchers have developed methods to remotely transfer electrical energy to the micro-robots, such as wireless power transfer (WPT) technology [18,19,20,21]. Wireless power transfer technologies can significantly enhance the usefulness of electronic devices or modules, and make them more practical and safer. WPT technology applications include microwave power transmission using gigahertz-frequency-range solar power satellites [22,23,24], radio-frequency identification (RFID) [25], energy harvesting, and magnetic resonant WPT which operate in the kilohertz and megahertz ranges [26,27,28]. The power transfer efficiency of gigahertz-frequency WPT systems are currently greater than 70% [22], while the power transfer efficiency in the kilohertz and megahertz ranges are in the range of 83%–90% [28]. Applications of magnetic resonant WPT technologies are now being actively developed by a number of electronics companies, as well [28]. This technology uses a low-impedance loop coil structure to generate an electromagnetic field, where the magnetic field is predominant. When the magnetic resonant WPT is employed in a micro-robot, power can be delivered from an external power source to a movable secondary load coil over relatively large air gaps. By utilizing WPT technology in this way, the micro-robot can wirelessly and continuously receive electrical energy. For instance, some studies [19,20] have adopted a motor system to provide propulsion to a micro-robot. However, these kinds of micro-robots are in the cm size range because of limitations in miniaturizing the motor system. Accordingly, these kinds of micro-robots have only been utilized as endoscope micro-robots. Additionally, due to the size of these robots, they have relatively small load coils than other electrical devices and modules. Thus, the power transfer efficiency of these micro-robot is around 1.6% [20].

To simplify the propulsion mechanism of micro-robot, recent studies have proposed the idea of generating propulsion force and torque with a WPT system while simultaneously transferring electrical energy [29,30]. However, the sizes of these devices have also been reported to be in the cm-scale range, which is much too large for medical purposes. For example, among the vessels of the cardiovascular system, which have various inner diameters, the vena cava, aorta and veins have 5–30 mm inner diameters [2]. This means that the size of an implantable micro-robot should be less than 5 mm. 

Accordingly, before micro-robots can be practically used for medical applications, further miniaturization is required. However, research to develop a mm-sized micro-robot generated propulsion force using a WPT system has not significantly advanced for several technical reasons.

A minimized WPT-system-based micro-robot would need to have an extremely small load coil. However, as the load coil size becomes smaller, the mutual inductance between the source coil and the load coil decreases dramatically. This phenomenon leads to diminished induced current; therefore, the propulsion force of the micro-robot is also reduced because the propulsion force is proportional to the induced current. This reduction in the propulsion of the micro-robot can be mitigated by determining the optimal load coil design structure that can generate the maximum propulsive force. 

In this paper, an efficient coil structure for generating a propulsion force and torque using WPT in a mm-sized micro-robot is proposed, using an analytical approach to consider structural and electrical factors. Furthermore, the fundamental concepts of WPT systems and the mechanism for generating a Lorentz force in a WPT coil, as well as the analytical formulations of the propulsion force and torque, are introduced. Based on these formulations, the important parameters that determine the magnitude of the Lorentz force were evaluated and, based on these considerations, an efficient coil structure and its dimensions were determined. The electrical aspects for generating the Lorentz force, such as allowable current, skin effect, and the number of turns in the coil were also considered to increase the induced current.

The experimental results showed that the fabricated 3 mm-sized micro-robot could achieve a velocity of 1.2 mm/s. A micro-robot of this size is meaningful, in that the proposed micro-robot is ten times smaller than those reported in previous research [30] involving electrical energy and propulsion force transfer.

## 2. Generating a Lorentz Force Using a Wireless Power Transfer System 

Since the proposed micro-robot is based on a WPT system, the fundamental concepts of WPT and the Lorentz force on a WPT system are briefly introduced in this section.

**Figure 1:**
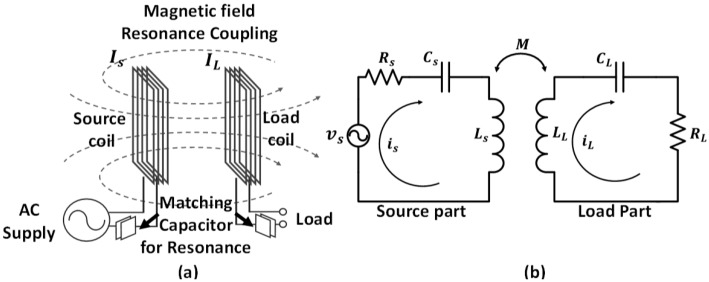
Concept of the WPT system: (**a**) the WPT mechanism and (**b**) equivalent circuit model.

A WPT system consists of two coils, a source coil and a load coil, as shown in Figure 1a. When a time-varying current flows in the source coil, it generates a time-varying magnetic field, and this magnetic field induces current in the load coil. In the equivalent circuit of the WPT system, the coupling of the source coil and load coil is described as mutual inductance, as shown in Figure 1b. Under this condition, the induced current in the load part can be derived using Equation (1). To maximize the electrical energy and transfer efficiency, both the source part and load part are designed with an LC resonant circuit so that the impedance of the inductance and capacitance can cancel each other out.

(1) $$I_{L}=\frac{j\omega M}{\left(R_{L}+j\omega L_{L}+\frac{1}{j\omega C_{L}}\right)}I_{S}\;$$

where $I_{L}$ and $I_{S}$ denote the phasor forms of the load coil current ($i_{L}$) and the source coil current ($i_{S}$), respectively. Here, $L_{L}$, $R_{L}$, and $C_{L}$, represent the inductance, resistance, and capacitance of the load coil, respectively, while M is the mutual inductance between the source coil and the load coil, and ω is the angular frequency.

In practice, to match the LC resonance, a controlling capacitor is used since the inductance of the oad coil is determined by the dimensions of the coil structure, and it is difficult to change its value [28].

Regarding the Lorentz force generated in one segment of the load coil in time-average, Equation (2) can be obtained:

(2) $$F_{avg}=\frac{1}{T}\int_{0}^{T}\left|B\right|\cos(\omega t)\left|I_{L}\right|\cos(\omega t+\alpha )l\;dt\;=\;2\pi \left|B\right|\left|I_{L}\right|\cos(\alpha )l$$

where *B*, $I_{L}$, α, and l represent the magnetic field generated by the source current flowing through the source coil, the induced current in the load coil, the phase difference between the source current and the load current, and the wire length of one segment of the load coil, respectively.

Based on Equations (1) and (2), although the maximum power transfer occurs in the LC resonance circuit, this type of WPT system cannot generate a Lorentz force because the phase difference between the source current and the load current is 90°. Therefore, it is necessary to control the phase between the source current and the load current. To generate a Lorentz force in the load coil the optimum phase difference between the source current and the load current is 45 degrees, according to the author [30]. 

## 3. Coil Design for Micro-Robot Miniaturization

To utilize a micro-robot as an implantable device, miniaturization is essential. However, a miniaturized micro-robot has a very small coil diameter as well as a small numbers of turns, which weakens the mutual inductance between the source coil and the load coil. Furthermore, because the Lorentz force is proportional to the length (*l*) in Equation (2), which is affected by the incident magnetic field, the minimization of the micro-robot or load coil leads to a reduction in propulsion force and torque. Therefore, to determine a design to generate greater propulsion force and torque, an analytical analysis was performed to identify the parameters that affect the Lorentz force.

In this section, modeling that was conducted to derive the propulsion force and torque in the coil is introduced with a dimensional analysis, which was performed to determine how the parameters influence the Lorentz force and torque.

### 3.1. Modeling of the Load Coil and Deriviation of Force and Torque

**Figure 2:**
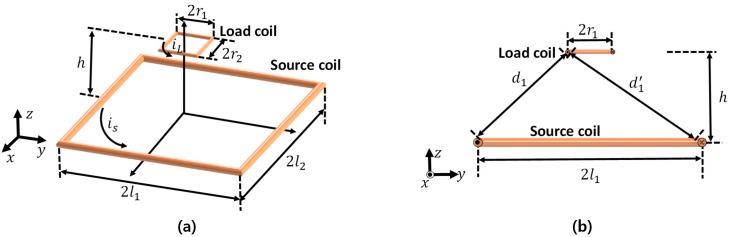
Two rectangular coils with wire $l_{1},\;l_{2}$ and $r_{1},\;r_{2}$ for analytical calculation of force and torque: (**a**) perspective view and (**b**) front view.

As shown in Figure 2, there are two coils in the WPT system, which generates the Lorentz force. Under this condition, the generated time-average force in the z-axis from all segments of the load coil can be derived with Equation (3):

(3) $$\begin{array}{l} \left|F_{avg\_z}\right|=I_{L}I_{S}\;\cos(\alpha )\;\frac{\mu_{0}h}{\pi }\;[\frac{r_{1}}{d_{1}^{\prime }{}^{2}}\;\left\{\frac{\left(l_{1}+r_{1}\right)}{\sqrt{{\left(l_{1}+r_{1}\right)}^{2}+d_{1}^{\prime }{}^{2}}}+\frac{\left(l_{1}-r_{1}\right)}{\sqrt{{\left(l_{1}-r_{1}\right)}^{2}+d_{1}^{\prime }{}^{2}}}\right\}-\frac{r_{1}}{d_{1}^{2}}\left\{\frac{\left(l_{1}+r_{1}\right)}{\sqrt{{\left(l_{1}+r_{1}\right)}^{2}+d_{1}^{2}}}+\frac{\left(l_{1}-r_{1}\right)}{\sqrt{{\left(l_{1}-r_{1}\right)}^{2}+d_{1}^{2}}}\right\}\\ \;\;+\frac{r_{2}}{d_{2}^{2}}\left\{\frac{\left(l_{2}+r_{2}\right)}{\sqrt{{\left(l_{2}+r_{2}\right)}^{2}+d_{2}^{2}}}+\frac{\left(l_{2}-r_{2}\right)}{\sqrt{{\left(l_{2}-r_{2}\right)}^{2}+d_{2}^{2}}}\right\}-\frac{r_{2}}{d_{2}^{\prime }{}^{2}}\left\{\frac{\left(l_{2}+r_{2}\right)}{\sqrt{{\left(l_{2}+r_{2}\right)}^{2}+d_{2}^{\prime }{}^{2}}}+\frac{\left(l_{2}-r_{2}\right)}{\sqrt{{\left(l_{2}-r_{2}\right)}^{2}+d_{2}^{\prime }{}^{2}}}\;\right\}]\; \end{array}$$

where $\;d_{1}\;=\;\sqrt{{\left(l_{2}-r_{2}\right)}^{2}+h^{2}}\;,\;d_{1}^{\prime }{}^{2}\;=\;\sqrt{{\left(l_{2}+r_{2}\right)}^{2}+h^{2}}\;,\;d_{2}\;=\;\sqrt{{\left(l_{1}-r_{1}\right)}^{2}+h^{2}}\;,\;d_{2}^{\prime }\;=\;\sqrt{{\left(l_{1}+r_{1}\right)}^{2}+h^{2}}$, $I_{L}$, $I_{S}$, and α represent the magnitudes of the source current and the load current, as well as the phase difference between the source current and the load current (α ≠ 90°).

**Figure 3:**
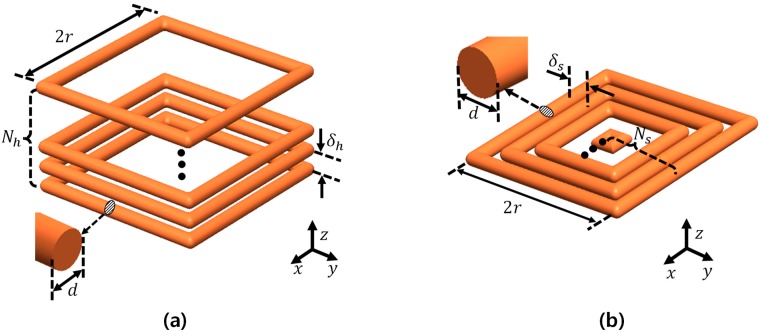
Dimensions of the load coils: (**a**) helical coil with a pitch of $\delta_{h}$; $N_{h}$ represents the number of turns, and (**b**) a spiral coil with the wire gap $\delta_{s}$, with the number of turns $N_{s}$.

By utilizing Equation (3), both the helical and spiral coils can be simplified into a stack of multiple single coils whose outermost length is 2*r*, separated by vertical distance $\delta_{h}$, and a combined co-axial rectangular coil with lateral distance $\delta_{s}$ respectively, as described in Figure 3.

For utilizing Equation (3), both coils can be simplified to a stacked single coil whose outmost length is 2*r*, in multiple with vertical distance $\delta_{h}$, and combined co-axial rectangular coil with lateral distance $\delta_{s}$, respectively, as described in Figure 3.

As Figure 3 illustrates, the reduced total coil wire length of spiral one compared to the helical structure is unavoidable since the wire length is smaller toward the inside of the coil. The total wire length of the helical coil and spiral coil with the number of turns $N_{h}$, $N_{s}$, respectively, in Figure 3 can be derived as follows:

(4) $$\begin{array}{l} L_{helical}\;=\;N_{h}\times (4\times 2r)=8N_{h}r\;\\ L_{Spiral}\;=\;4N_{s}\times \{2r-(N_{s}-1)(2d+\delta_{s}\} \end{array}$$

where $N_{h}$, $N_{s}$, and *d* represent the number of turns in the helical coil and the spiral coil, as well as the diameter of each coil wire, respectively. 

For practical implementation, in this research the dimensions of the micro-robot were assumed to be 2*r* × 2*r* × 2*r*. Then, the maximum numbers of turns in the helical coil ($N_{h}$) and the spiral coil ($N_{s}$) can be evaluated as follows:

(5) $$\begin{array}{l} N_{h}=\;\frac{2r}{d+\delta_{h}}\;\\ N_{s}=\;\frac{r}{d+\delta_{s}}\; \end{array}$$

Equation (6) represents the *z*-axis Lorentz force ratio generated in the load coil according to the number of turns and wire gap. The most important factor that introduces the difference between the two forces is the total wire length, which is represented by *r* in Equation (4). 

(6) $$F_{helical}\;:\;F_{spiral}\;=\;N_{h}r\;:\;N_{s}r-\frac{N_{s}(N_{s}-1)}{2}\delta_{s}\;=\;4r\;:\;r+d+\;\delta_{s}$$

If the helical and sipiral coil structures have the same outer radius, wire diameter (*d*), and wire gap ($\delta_{h}=\delta_{s})$, then the Lorentz force on the helical coil is nearly four-times greater than on the spiral coil, since the diameter of the wire (*d*) and wire gap ($\delta_{s}$) are negligibly smaller than the coil size (*r*). 

**Figure 4:**
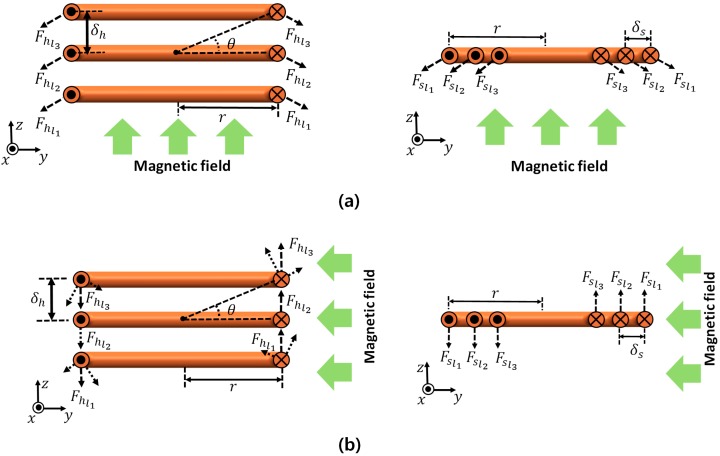
Generated force and torque according to an incident magnetic field vector. (**a**) When the source coil and load coil are in parallel positions, propulsion force is generated. (**b**) When the source coil and load coil are in perpendicular positions, torque is generated on the load coils. (**a**) Helical coil with pitch $\delta_{h}$ and (**b**) spiral coil with wire gap $\delta_{s}$.

When the incident magnetic field is perpendicular to the load coil, it generates a torque due to the Lorentz force, as shown in Figure 4b. Based on its coil structure type, both coils have different characteristics. In the case of the helical coil, the generated torque can be described by:

(7) $${\sum \tau }_{h}=2\sum_{i=1}^{N_{h}}\left[\left\{F_{h_{l(i)}}\cos(\theta_{i})\right\}\cdot \frac{r}{\cos(\theta_{i})}\right]\;=4BiN_{h}r^{2}$$

where the $N_{h}$, $F_{h_{l\left(i\right)}}$, *r*, and *B*, denote the number of turns in the coil, the generated Lorentz force in one segment of the coil wire in the *i*th loop, the radius of one single coil, and the magnetic field that is incident from the source coil, respectively. In the case of the spiral structure coil, the generated torque can be described.

For the spiral structure coil, the generated toque can be described by:

(8) $$\sum \tau_{s}=2\sum_{i=1}^{N_{s}}\left[F_{s_{l(i)}}(r-\delta_{s})\right]\;=4BiN_{s}\left\{r^{2}-(N_{s}-1)r\delta_{s}+\frac{(N_{s}-1)\delta_{s}{}^{2}}{6}\right\}\;$$

where the $N_{s}$, $F_{h_{s\left(i\right)}}$, *r*, $\delta_{s}$, and *B* denote the number of turns in the coil, the generated Lorentz force in one segment of the coil wire in the *i*th loop, the radius of one single coil, the wire gap in the spiral load coil, and the magnetic field from the source coil, respectively. 

(9) $$\tau_{s}\;:\;\tau_{h}\;=\;2r^{2}\;:\;r^{2}-\left(\frac{r}{d+\delta_{h}}-1\right)\delta r+\;\left(\frac{r-d-\delta_{h}}{6(d+\delta_{h})}\right)\delta^{2}$$

Comparing the torque in each coil having the same outer radius, wire diameter, and wire gap ($\delta_{h}=\delta_{s})$, and based on Equations (4), (5), (7), and (8), the torque ratio between the helical coil and the spiral coil can be obtained as Equation (9). Since the diameter of the coil wire (*d*) and the wire gap ($\delta_{s},\delta_{h}$) are smaller than the coil size (*r*), the spiral structure generates two-times greater torque than the helical coil.

### 3.2. Analysis of Design Parameters on Load Coil 

Since the Lorentz force is proportional to the induced current, one way to increase the Lorentz force is to increase the induced current. The induced current is determined by the operating frequency and the mutual inductance of the load and capacitors. Since the small micro-robot receives only limited power, the losses of small components should be considered. Therefore, Equation (1) should be modified into Equation (10), which considers the inner resistance in the load coil:

(10) $$I_{L}\;=\;\frac{j\omega M}{R_{L}\;+\;\left\{j\omega L_{L}+\frac{1}{j\omega C_{L}}+R_{L}+R_{in}\right\}}I_{S}\;=\frac{j\omega M}{R_{L}\;+\;\left\{j\omega L_{L}+\frac{1}{j\omega C_{L}}+R_{L}+\frac{\rho L}{\pi (\delta_{sk}d-\delta_{sk}{}^{2})}\right\}}I_{S}\;$$

where $I_{L}$, $I_{S},\;\;M,\;R_{L},\;L_{L},C_{L},\;\;\rho ,\;\;L,\delta_{sk}$, and d denote the induced current in the load coil, the source current, the angular frequency, the mutual inductance between the source coil and the load coil, the resistance of the micro-robot, the inductance of the load coil, the capacitance of the load coil, the resistivity of the load coil, the total wire length of the load coil, the skin depth of the load coil, and the diameter of the load coil wire, respectively.

**Figure 5:**
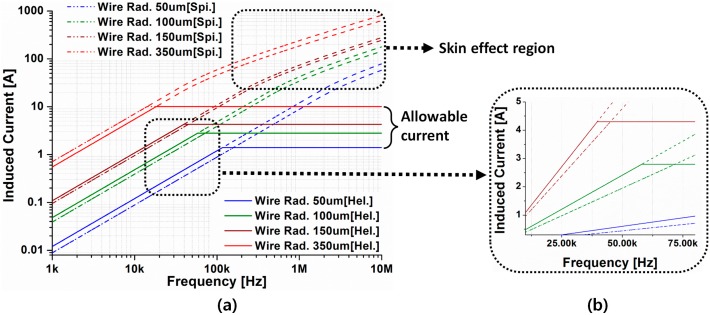
Induced current with 3 mm × 3 mm load coil in relation to the frequency under allowable current (**a**), and figure showing the different in slope (**b**).

As shown in Equation (10), although the load current looks proportional to the frequency, the allowable current in the μm-sized wire diameter limits this characteristic, as shown in Figure 5. Moreover, as the frequency increases, the skin effect occurs; therefore, the inner resistance of the load coil increases. The non-linear part of Figure 5 illustrates this phenomenon. Therefore, considering the skin effect, the operating frequency should be lower than the MHz range under the μm size so that the wire diameter of the load coil is small enough ($\delta_{sk}\gg d$) that the skin depth is negligible.

Additionally, the induced current is proportional to the mutual inductance between the source coil and the load coil. Since the mutual inductance is proportional to the cross-section area affected by the incident magnetic field, it is usually determined by the number of turns and the coil radius. In this research, the outermost radius was assumed to be restricted; thus, the valid factor in determining the mutual inductance is the number of turns in the load coil, as seen in Section 3.1, Equation (10). 

Both the spiral and helical coil wires have an allowable current related to the wire diameter. In general, as the wire diameter increases, the allowable current also increases, as seen in Figure 5. However, excessive wire diameter is inefficient because it diminishes the number of turns in the load coil based on Equation (5).

The wire gap between the wire in the load coil is influenced by the total length of the load coil, which affects the inner resistance of the load coil. As the wire gap increases, the number of turns in each coil structure decreases, and the mutual inductance between the source coil and the load coil decreases. Furthermore, as the total length of the load coil shortens, the incident magnetic field length (*l*) in Equation (2) shortens. This phenomenon reduces the generated Lorentz force and torque in the load coil. Therefore, the wire gap should be zero or it should be reduced as much as possible.

**Figure 6:**
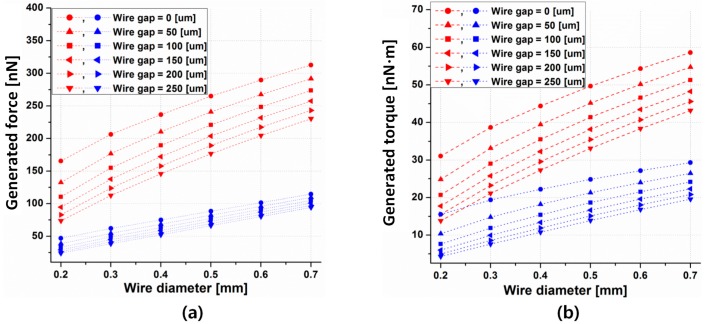
Simulated force (**a**) and torque (**b**) under the allowable current according to the wire diameter of the load coil.

Figure 6 shows the propulsion force and torque simulation results according to the wire diameter and wire gap. Each case has the maximum allowable current based on the coil wire diameter in both the helical and spiral coils. Thus, since the wire with a larger diameter has a larger allowable current, both propulsion force and torque increase as the wire diameter is increased. Apparently, the propulsion force and torque obtained on the helical coil is greater compared to the spiral one because of the total length differences. Even with the same number of turns, wire gap, and wire diameter, the propulsion force and torque in the helical coil is about four-times and two-times higher than the spiral coil, respectively. These results are supported by Equations (5) and (6). 

However, because of the difficulties involved in fabricating a multi-layer helical coil, it can be substituted by using a spiral coil with a greater number of turns and a smaller wire diameter. For example, a spiral coil with a 0.4 wire diameter and 0 wire gap is easier to fabricate than a helical coil with a 0.2 wire diameter and a 250 μm wire gap. Thus, depending on the coil structure and coil dimensions, that is, the wire diameter, wire gap, operating frequency, and number of turns in the load coil, the Lorentz force and torque can be changed even while maintaining an identical outer radius of the load coil.

## 4. Experimental Verification

**Figure 7:**
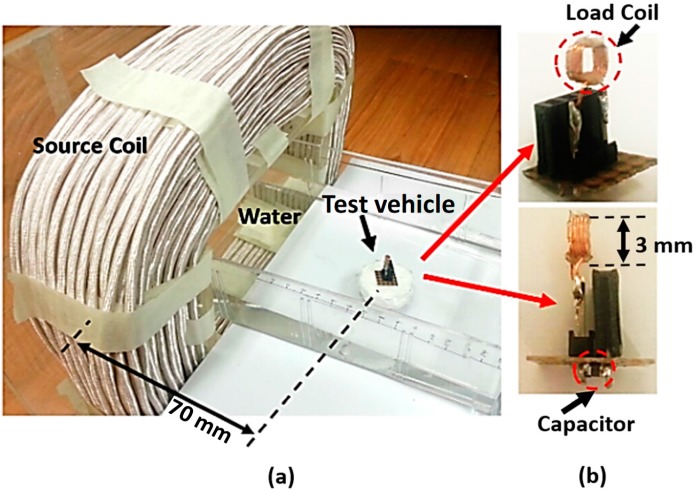
Photographs of the experimental setup (**a**) including source coil, load coil (helical), and the test vehicle floating on water; and (**b**) the load coil with a helical structure.

A micro-robot with a wirelessly powered load coil was implemented to experimentally verify the proposed formulation and the simulation results of the propulsion force and torque generation, for a 111-turn rectangular coil carrying a 2.1-A current with an 87 kHz frequency. To reduce the resistance of the source coil, a Litz wire was used for the coil winding of the source coil. The dimensions of the fabricated coils are shown in Figure 7, and the detailed electrical parameters of the coils (3 mm load coil diameter with 0.05 mm wire gap) are listed in Table 1. The load coil was fabricated with solid copper wire. For a more precise comparison of the effect of the structure, the induced current was measured and maintained at 0.5 A for each case by changing the resistors. 

The magnetic field generated by the source coil was able to reach a square-shaped load coil located 70 mm away from the source coil, as shown in Figure 7. The energy transfer efficiency was 1.07% in the 10-turn spiral load coil, and the efficiency varied by around 1%–2% depending on the various load coils used in this experiment. This is a relatively low power transfer efficiency for WPT applications in general [22,23,24,25,26,27,28]. However, in fact, the power transfer efficiency in implantable micro-robots and devices in previous studies are not that high [31,32]. One reason for the low efficiency is that the mutual inductance in this system is low because of its tiny load coil. Another reason for the energy transfer reduction is the lack of LC resonance, which is necessary to generate a Lorentz force in the load coil.

**Figure 8:**
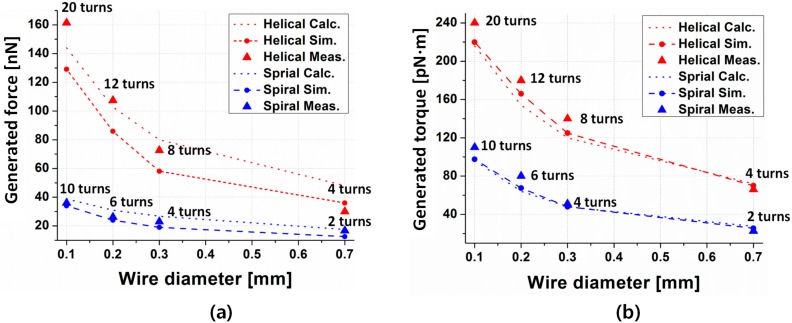
Analytical calculation, numerical simulation and measurement results of propulsion force (**a**) and torque (**b**) in relation to the wire diameter with a 3 mm load coil.

In the experiment to generate propulsion and torque, various capacitors were connected in each case to the load coil to control the phase difference between the source current and the load current, so that it was 45 degrees, as shown in Figure 8b. Especially in the case of torque generation, the angle between the incident magnetic field and the normal vector of the load coil was set at 45 degrees.

To verify the propulsion force and torque that was generated, the micro-robot with the load coil was fixed onto a small test vehicle floating on water using extruded polystyrene foam (Styrofoam), as shown in Figure 8a. The test vehicle design was a cylinder type 40 mm in diameter and 15 mm in height to minimize the resistance of the water effect during the rotational experiments. The weight of the extruded polystyrene foam alone was 3 g, and total weight of the test vehicle was around 4 g; it can change depending on the coil and number of capacitors. However, because a surface-mount device (SMD)-type capacitor is used, their effect on weight of the test vehicle is negligible. Additionally, to prevent random movement of the test-vehicle, the experiments were performed after the test vehicle was stabilized. 

The motions of the test vehicle were recorded using a high-speed video camera, and the velocity and angular velocity were precisely measured, using frame-by-frame analysis of the recorded video. All of the frames were marked with the location of each pixel of the test vehicle, and then the differences between the pixel locations were converted to real distance values. Since the frame differences imply a time variation, we could obtain total travel distance and travel time data using the video capture. Finally, the time-average velocity of the test vehicle was obtained.

Once the velocity and angular velocity of the test vehicle were measured, they could be converted into a propulsion force and torque using the following equations, respectively [33,34]:

(11) $$F_{prop}=\;\frac{1}{2}c_{D}\rho_{w}V^{2}S\;$$

(12) $$\tau =\;\frac{1}{2}c_{D}\rho_{w}\;\omega^{2}Sr\;$$

In Equation (11), $F_{prop}$ represents the propulsion force of the micro-robot, *C_D_* denotes the drag coefficient of the test vehicle during the propulsion force experiment, $\rho_{w}$ denotes the density of water, *V* denotes the velocity of the micro-robot, and *S* denotes the wetted area of the test vehicle. In this experiment, the wetted area was the same value for both the helical structure type and the spiral structure type because of the small volume.

Equation (12) describes the relationship between torque and angular velocity in the fluid conditions referenced in [34,35]. Here, *r* is half of the load coil size. The same types of extruded polystyrene foam test vehicles were used for the propulsion generation and torque generation experiments, as indicated in the experimental parameters listed in Table 2 [34]. 

The experimental results shown in Figure 8 demonstrate that the propulsion force in each coil varies with changes in its structure. Under the same induced current, the most dominant factor determining the propulsion force and torque in this research was the number of turns. For the helical coil, the maximum micro-robot propulsion force was measured to be 162 nN with a velocity of 1.2 mm/s. For the spiral coil, the maximum propulsion force was measured to be 38 nN at a velocity of 0.6 mm/s. 

Additionally, as the diameter of the wire increases, the number of turns in the coil decreases, which reduces the total length of the coil, eventually resulting in a decrease in propulsion force and torque for both the helical and spiral coil. The propulsion force and torque in the helical coil were four-times and two-times larger than in the spiral coil, as shown in Figure 8, which is highly consistent with the results of Equations (6) and (9).

## 5. Conclusions

We analyzed and compared two types of coil structure, which were used to generate both a propulsion force and receive electrical energy, respectively, using wireless power transfer (WPT) technology in a micro-robot. The magnetic force and torque between the source coil and the load coil were derived analytically and verified by numerical simulation and experimental measurement. Additionally, with respect to the electrical power transfer, the design parameters affecting the induced current, considering the allowable current, were evaluated as well. As a result, it can be concluded that the most advantageous design is to select a conductor with a radius smaller than the skin depth and to wind as many turns as possible. Furthermore, it was advantageous to select a maximum frequency within a range where the induced current did not exceed the allowable current. Depending on the structure of the coil, it was confirmed that the magnitude of the propulsion force and torque was up to four-times and two-times greater, respectively. Moreover, to obtain the maximum propulsion force and torque, we minimized the wire gap in the coils and chose the minimum diameter that could produce the allowable current required for the micro-robot. Based on this design, the fabricated 3-mm sized helical structure micro-robot achieved a velocity of 1.2 mm/s. These results are meaningful in that the micro-robot was ten times smaller than the conventional WPT-based micro-robot in the literature. This research can contribute to the further miniaturization of micro-robots.

## Figures and Tables

**Table 1:** Electric parameters of the WPT source coil and load coils.

Coil Type	Number of Turns	Wire Diameter (mm)	Self Inductance (μH)	Mutual Inductance (μH)	Resistance (ohm)
Source coil	111	-	3648	-	0.3
Load coil (Helical)	20	0.1	2.39	0.501	0.61
	12	0.2	0.753	0.372	0.12
	8	0.3	0.987	0.298	0.05
	4	0.7	0.390	0.142	0.02
Load coil (Spiral)	10	0.1	0.478	0.253	0.216
	6	0.2	0.431	0.193	0.07
	4	0.3	0.394	0.169	0.03
	2	0.7	0.313	0.135	0.015

**Table 2:** Experimental parameters of the test vehicle.

Parameters	Value
Drag coefficient ($C_{D}$)	0.64
Wetted area (*S*)	342.1 ${\mathrm{mm}}^{2}$
Density of water (ρ)	1000 $\mathrm{kg}/m^{3}$
Half wire length (*r*)	20 mm
